# Novel technique for arterial reconstruction in simultaneous pancreas-kidney transplantation, a randomized clinical trial

**DOI:** 10.1186/s13104-023-06568-9

**Published:** 2023-10-26

**Authors:** Hassan Karar, Mojtaba Shafiekhani, Mohamad Mahdi Mahmoudi, Nazanin Azadeh, Alireza Shamsaeefar, Sahar Sohrabi Nazari, Mehran Jafari, Kiarash Ashrafzadeh, Maryam Esmaeili, Saman Nikeghbalian, Hamed Nikoupour

**Affiliations:** 1 Shiraz Transplant Center, Abu-Ali Sina Hospital, Shiraz University of Medical Sciences, Shiraz, Iran; 2 Department of Hepatobiliary and Transplant Surgery, The National Center of Gastrointestinal and Liver Disease, Ibn-Sina Specialized Hospital, Khartoum, Sudan; 3 Transplant Research Center, Shiraz University of Medical Sciences, Shiraz, Iran

**Keywords:** Insulin-dependent Diabetes Mellitus, Simultaneous pancreas kidney transplantation, Pancreas Surgery

## Abstract

**Introduction:**

Simultaneous pancreas kidney (SPK) transplantation is an invaluable procedure to enhance the quality of life of insulin-dependent patients with advanced renal disease. The creation of vascular anastomoses of the donor’s pancreas vessels to the recipient’s, is of utmost importance to predict the graft outcome and surgical complications. In the study we introduce a novel technique for arterial reconstruction during SPK transplantation.

**Methods:**

Conventionally, during the SPK transplantation, a so-called Y-graft is anastomosed between donor’s superior mesenteric and splenic artery to the recipient’s right iliac artery. In the study we adopted a new technique by preparing an extra extension using the donor’s carotid artery, to be anastomosed to the Y-graft and the iliac artery. In this non-blinded randomized clinical trial we compared the surgical complications and early outcomes between the 2 groups of patients with the traditional and new arterial reconstruction techniques during 3 months after transplantation.

**Results:**

Thirty adult patients were included in the study. The incidence of pancreatitis, vascular thrombosis and surgical site infection was lower in the new Y-graft and extension technique, which was not statistically significant. However, the calculated Cohen’s d index showed the medium effect of new Y-graft and extension technique on complication after SPK transplantations.

**Conclusion:**

The post-operative complications tend to be lower in the novel arterial reconstruction technique, however a study on a larger patient group is encouraged to confirm our primary results.

**Trial registration:**

The study was registered at the Iranian Registry of Clinical Trials on 12/05/2022; IRCT 20210625051701N2; (http://www.irct.ir/).

## Introduction

In 1966, university of Minnesota performed the first pancreas transplantation as a physiologic treatment of diabetes mellitus (DM). Ever since, improvements of surgical techniques and introduction of new immunosuppressant therapies have helped the procedure gain more desirable outcomes [1]. Pancreas transplantation is more commonly performed at the same time with a kidney transplant, a procedure referred to as simultaneous pancreas-kidney (SPK) transplantation. SPK transplants are considered in patients with insulin-dependent DM and chronic renal failure requiring hemodialysis, either presently or potentially in the near future. The candidates might suffer progressive microvascular involvement and repeated hospitalizations for diabetes complications including diabetic ketoacidosis [2]. Less than 10% involve pancreas transplantation alone (PTA). 30% of those given a PTA will eventually need a kidney transplant because of the adverse cumulative effects of immunosuppression with calcineurin inhibitors [3].

Despite over two decades of experience and considerable refinements in the surgical techniques, the operation still carries a high morbidity. The majority of technical injuries to the pancreas result from pancreas devascularization during the combined liver-pancreas procurement [4]. The arterial supply of pancreas is divided into two gross regions. The pancreatic tail and body is mainly supplied by the dorsal pancreatic artery (DPA) and greater pancreatic artery, both of which are derived from the splenic artery [5]. The pancreas head and body region is supplied by the anterior superior (ASPDA) and posterior superior pancreaticoduoenal (PSPDA) arteries, as well as the inferior pancreaticoduodenal (IPDA) artery [6]. ASPDA and PSPDA are branches of the gastoduodenal arteries, which is derived from the common hepatic artery (CHA) [7]. Since liver is a vital “life-saving” organ whereas pancreas is considered as a “quality of life enriching” organ, during the simultaneous liver-pancreas harvesting, the transplant team takes the aortic patch containing the CHA and divides the gastroduodenal and splenic arteries [8]. Chances are that during the procedure, the DPA and IPDA get injured, rendering the organ prone to ischemia and arterial thrombosis [9].

The Abu Ali Sina solid organ transplantation hospital is a major center of its type in the Asia, performing more than 500 annual solid organ transplants, including pancreas [10]. In this study, we introduce a new arterial reconstruction technique in the SPK procedure aiming to enhance the arterial graft which reduces the vessel end-to end anastomosis time, pancreatic manipulation and postoperative complications.

## Methods

### Trial design and patient population

**Fig. 1:**
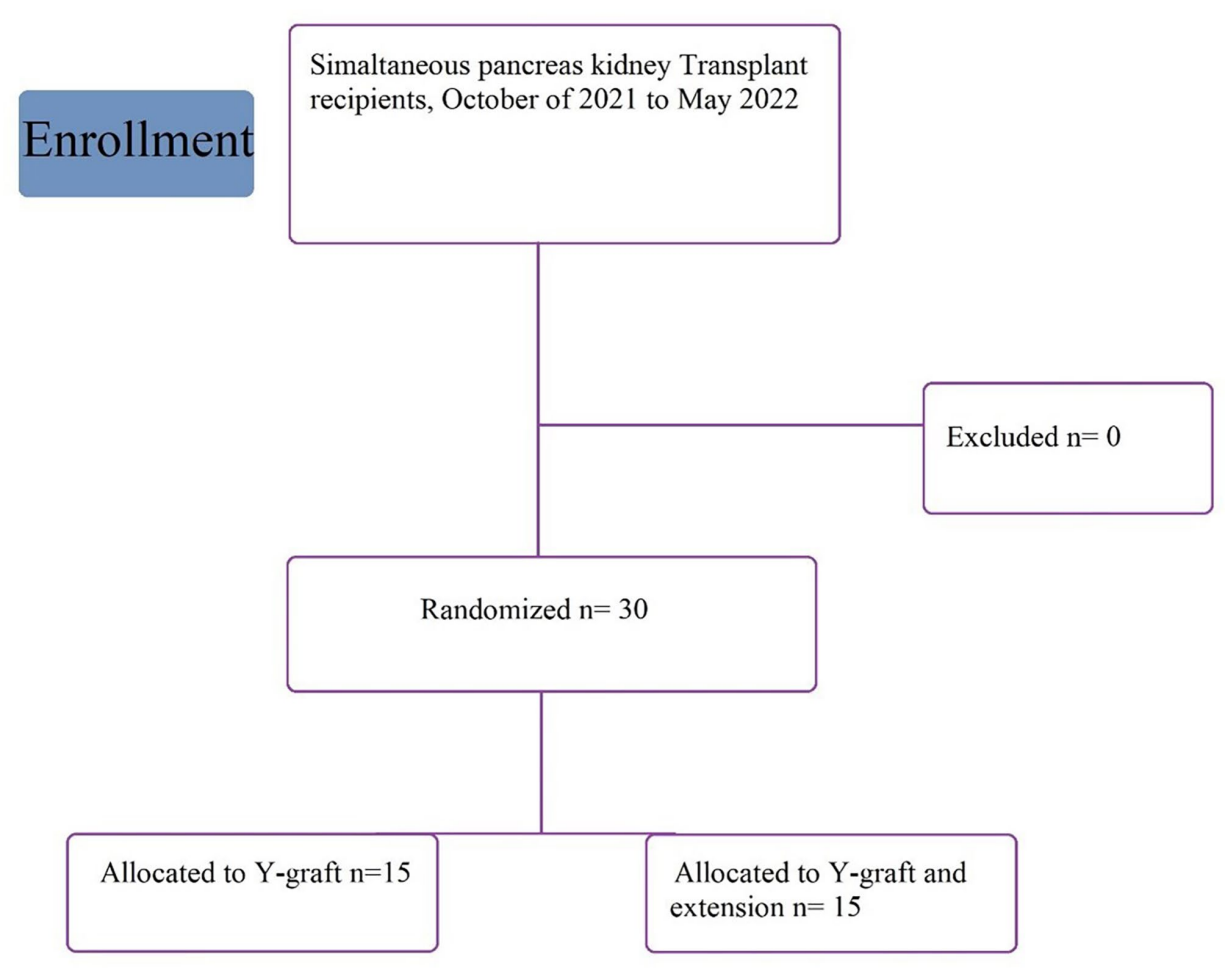
CONSORT diagram of the enrollment, inclusion and exclusion of patients in the course of study

The study was conducted as a non-blinded randomized clinical trial in the Abu Ali Sina solid organ transplantation hospital which affiliated to Shiraz University of Medical Sciences, Shiraz, Iran from July of 2022 to December of 2022. The simple randomized method was used to randomize the study subjects. The participants included those of 18 years old or above, suffering from diabetic nephropathy who met the SPK criteria of our center [10]. The donors were selected among the dead-brain patients less than 40 years old with creatinine level of less than 2 mg/dL who did not require inotropes or required only low inotrope doses. As the recipients, the diabetic nephropathy patients who underwent portal venous drainage were included in the study; whereas those who underwent pancreatic systemic venous drainage were excluded from the study (Fig. 1). In some patients, mostly due to adhesions of previous surgeries, the SMV can not be found. In such cases, we have to approach the systemic method and therefore, there is no need for extension graft. High BMI might also contribute to difficulty finding the SMV. Whatever the cause is, patients who underwent the systemic method were excluded from the study.

The protocol and patient informed consent form were reviewed and approved by the local Ethics Committee of Shiraz University of Medical Sciences (IR.SUMS.MED.REC.1401.045). The study was registered at the Iranian Registry of Clinical Trials (IRCT 20210625051701N2; http://www.irct.ir/).

### Surgical intervention and techniques

**Fig. 2:**
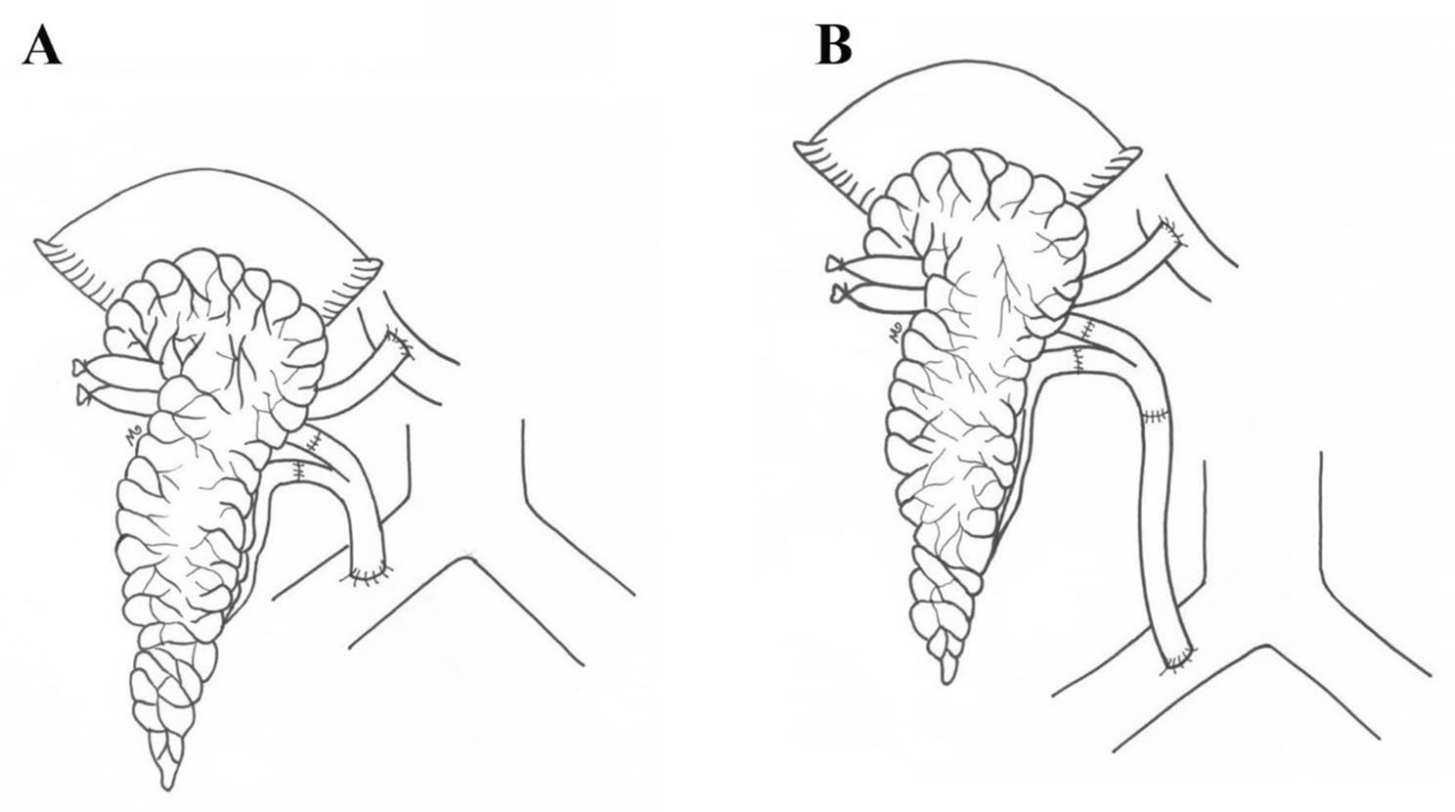
The schematic depiction of the Y-graft technique (A) and the Y-graft and extension technique (B)

In this study, we clamped the superior mesenteric vein (SMV) to the root of the mesentery and after SMV venotomy, created an end-to-side portal vein to SMV anastomosis with the standard vessel anastomosis technique. For arterial reconstruction the patients were randomized to two groups: we used either the Y-graft technique (the classical technique commonly used in our center) or the Y-graft and extension technique (the new technique to be investigated here). The arterial reconstruction in the Y-graft technique is yielded by the anastomosis of the Y-graft to the common iliac artery (CIA) (Fig. 2-A).

In the new technique of Y-graft and extension, the Y-graft is created with short limbs and one extension graft is prepared, using the carotid to be anastomosed to the CIA (Fig. 2-B). Therefore, there is no need to explore the recipient to decide the appropriate type of bench surgery. Furthermore, the occurrence of arterial thrombosis, kinking and tension is less probable compared to the traditional technique of Y-graft. In the Y-graft and extension technique the anastomosis is created while preparing the graft and is passed through the intestinal mesentery; therefore, the implantation time remains intact and graft manipulation is decreased.

In the study, we created portal venous drainage for outflow of the pancreas. Patients were randomly selected for either of the arterial anastomosis method. For the first group, the Y-graft was directly anastomosed to the right iliac artery and for the second group an extension artery was prepared using the carotid artery. The extension was then, crossed through the mesentery and anastomosed to the right iliac artery, close to SMV. For all patients, pancreas was transplanted prior to the kidney. According to our center guidelines, the kidney is transplanted via the same abdominal incision, in the retroperitoneum and on the left side.

During the surgery we did not close the fascia and leave it to be done in the following one year. Patients underwent simultaneous appendectomy and they were transferred to the post-transplantation intensive care unit with 2 drainage tubes, one inserted near the kidney and one near the pancreas. All the patients were transferred to the organ transplantation intensive care unit after the operation. Methylprednisolone (1 g/day till 3 doses) and ATG (1.5 g/day till 4 days) were used as induction immunosuppression and then anti metabolites, CNIs and prednisolone were used as maintenance therapy. The desired Tacrolimus blood level is 8–12 ng/dL which was first checked on the 3rd day. Patients were then extubated as soon as possible.

The procedure of SPK transplantation, similar to any major surgeries, carries a high risk of thromboembolism. The rotational thromboelastometry (ROTEM) test was requested for the patients, if the result showed a hypercoagulable state, anticoagulation with heparin was started during hospitalization and patients were discharged with Aspirin. But if it shows hypocoagulative or normal state, we do not give any anti-coagulants either during hospitalization or after discharge.

### Patient follow up and data collection

**Fig. 3:**
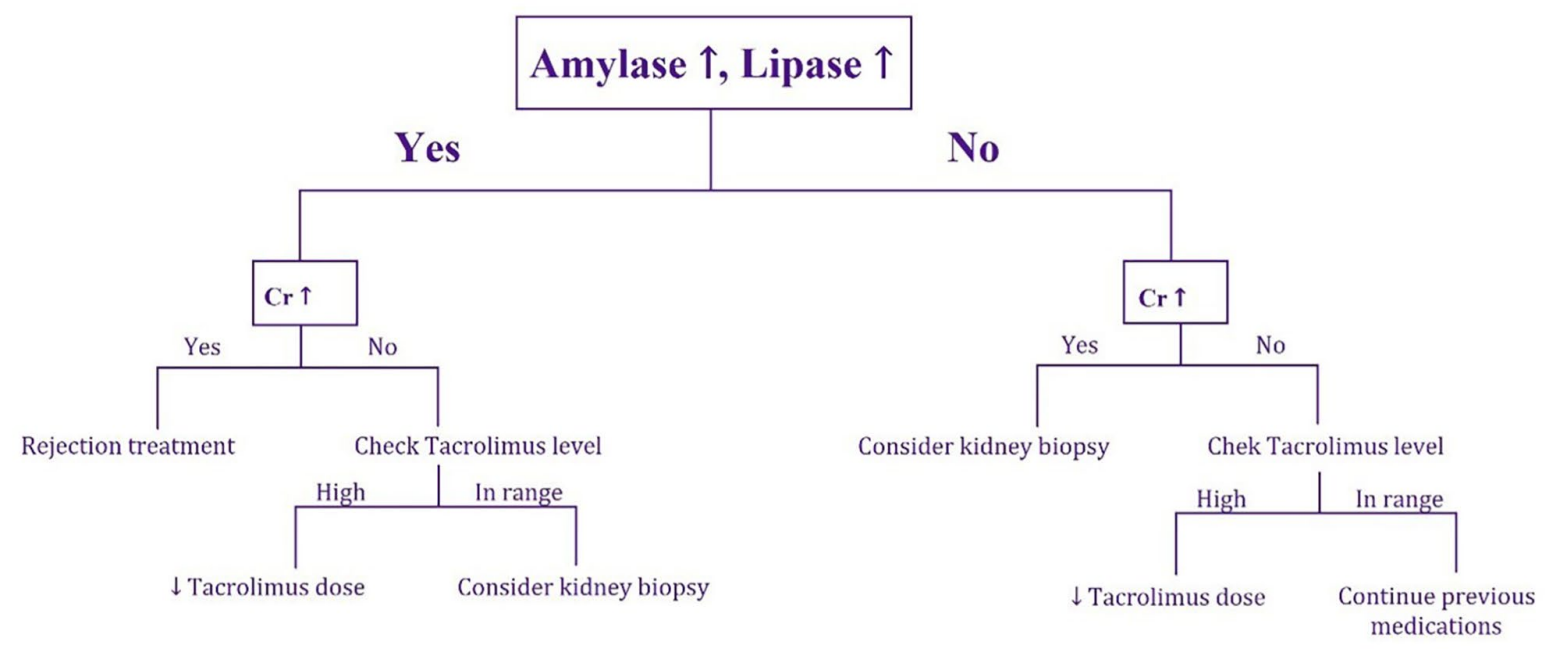
The algorithm of diagnosis and management of suspicious rejection episodes among Simultaneous pancreas-kidney Transplant recipients

Patients were visited by a multidisciplinary team including a nephrologist, the transplant surgeons and a clinical pharmacist on a daily basis for clinical evaluation and immunosuppressive adjustment. The daily renal function tests, blood sugar chart and pancreatic enzymes were meticulously evaluated. An abdominal ultrasound, as well as color Doppler evaluation of the graft vessels, were demanded every other day. Patients were weekly visited by a nephrologist and a transplant surgeon after being discharged from the hospital. Any complications or suspicious rejection were immediately referred to the hospital for further assessment and management. Any suspicious Rejection episode was diagnosed as the following algorithm (Fig. 3.). The patients’ data were accessed by following their electronic documents and all of them had been followed within 3 months after transplantation.

### Clinical outcomes

The current study is conducted to compare the incidence of the post-surgical complications including pancreatitis, vascular events, fistula formation, and rejection between the two methods in the first 3 months following the transplantation.

### Statistical analysis

Categorical variables were described as frequency rates and percentages, and continuous variables were described using mean, median, and interquartile range (IQR) or standard deviation (SD) values. Means for continuous variables were compared using the Mann-Whitney test. Proportions for categorical variables were compared using the χ2 test, and the Fisher exact test was used when the data were limited. To calculate the quantitative measure of effect size, the Cohen’s d index was measured for the qualitative outcomes of the two methods using mean and standard deviation. The Cohen’s d index of less than or equal to 0.2 was considered as a small size effect, whereas 0.5 and 0.8 results were considered as the medium and large size effects, respectively. All statistical analyses were performed using SPSS (Statistical Package for the Social Sciences), version 16.0 software (SPSS Inc). A P-value of lower than 0.05 in the multivariate analysis was considered significant.

## Results

A total number of 30 patients were included in the study. The patients were divided to 2 equal groups of 15, one group had underwent the Y-graft technique and for the other one the Y-graft and extension technique was applied. The mean age of patients was 33.1 ± 6.3 years. Among the patients, 19 were male and 11 were female. Table 1 shows the demographic data of the study population.

**Table 1:** The demographic and clinical characteristics data of the Simultaneous pancreas-kidney Transplant recipients (N = 30)

Variants	Total (N = 30)	Y-graft method (N = 15)	Y-graft and extension method (N = 15)	p-value
Age (year)	33.1 ± 6.3	34.6 ± 6.3	31.5 ± 6.1	0.78
Sex				
Male Female	19 11	7 8	12 3	0.64
BMI (Kg/m^2^ ) (recipient)	21.4 ± 3.4	21.4 ± 4.3	21.3 ± 2.4	0.096
BMI (Kg/m^2^ ) (donor)	20.00 ± 6.98	20.22 ± 7.43	19.91 ± 6.49	0.71
Donor age (year)	25.3 ± 8.7	24.5 ± 7.1	26.1 ± 10.3	0.086
Duration of DM (year)	19.1 ± 4.2	19.6 ± 4.4	18.5 ± 4.1	0.799
Duration of ESRD (year)	22 ± 7.6	23.2 ± 8.1	20.8 ± 7.1	0.52
Type of dialysis				
HD PD	27 3	14 1	13 2	0.50

Abbreviations: Body Mass Index (BMI), Diabetes Mellitus (DM), End-Stage Renal Disease (ESRD), Hemodialysis (HD), Peritoneal Dialysis (PD).

Table 2. shows the clinical outcomes of both techniques. In the Y-graft and extension technique, the occurrence of venous thrombosis, pancreatitis, rejection and surgical site infections was lower; however none of them were statistically significant. As it is demonstrated in Table 2. The size effect for pancreatitis, venous thrombosis, graft rejection and operation time was calculated as medium which indicates a larger sample size might show a statistically significant difference in the outcomes of the two techniques. The operation time was relatively shorter in the Y-graft and extension technique. Furthermore, for the 15 patients with the novel arterial reconstruction technique no organ failure or mortality was recorded during the follow up period.

**Table 2:** The occurrence of post-surgical complications of simultaneous pancreas-kidney Transplantation (N = 30)

Clinical outcomes	Total (N = 30)	Y-graft (N = 15)	Y- graft and extension (N = 15)	Cohen’s d index	p-value
Pancreatitis	4	3	1	0.62	0.29
Venous thrombosis	2	2	0	0.57	0.07
Arterial thrombosis	0	0	0		
Interoperation bleeding	8	5	3	0.29	0.34
Length of operation (hr)	4.65 ± 0.44	4.87 ± 0.32	4.42 ± 0.43	0.61	0.60
CIT (min)	187 ± 32	188 ± 30	185 ± 41	0.55	0.19
Rejection	6	4	2	0.58	0.32
Leakage of anastomosis site	2	2	0	0.30	0.24
Gl bleeding post SPK	16	10	6	0.42	0.14
Surgical site infections	5	3	2	0.55	0.50

Abbreviations: Gastrointestinal (GI), Simultaneous pancreas-kidney (SPK), Cold ischemic time (CIT)

## Discussion

Pancreas transplantation is mostly done simultaneously with a kidney transplantation (SPK). In this study we have introduced a new arterial anastomosis technique, in which an extension is prepared using the carotid artery. The extension is then anastomosed between the conventional y-graft and the right iliac artery. Preferably, pancreas is transplanted prior to the kidney to reduce the duration of cold ischemic time and reduce the risk of consequent graft failure [11]. Organ procurement is done at the same time as the recipient’s anatomy is being explored. In our center, we prioritize the portal venous drainage of pancreas over the systemic drainage [12]. In cases of difficult SMV exploration, we adopt the systemic venous drainage via anastomosing the donor veins to the recipient’s inferior vena cava (IVC).

In the Y-graft technique, intestinal wall edema may occur due to SMV clamping and there is possibility of difficulty closing the abdominal wall. Furthermore; sometimes the Y-graft reaches the CIA difficultly, creating some tension. To avoid this tension, the splenic arm and superior mesenteric artery (SMA) are cut longer, which could contribute to kinking of the arteries and increased risk of thrombosis. In the common Y-graft technique, it is not possible to estimate the length of the Y-graft until the recipient’s anatomy has been explored, which sometimes inevitably lengthens the cold ischemic time. Therefore, in the Y-graft and extension technique we prepare the graft with an extra arterial extension to decrease the cold ischemic duration and as stated, the duration of operation tends to be shorter. For venous outflow, we used to prepare the graft for the portal drainage technique rather than the systemic one, this would result in a long Y-graft which might be kinked, increasing the risk of thrombosis [13]. In contrast, in the Y-graft and extension technique, we prepare the graft for the systemic venous drainage. During the organ transplantation, if the recipient’s anatomy is desirable for the portal drainage, a conduit is made out of donor’s carotid artery and is anastomosed to the internal iliac vein. Consequently, both arterial and venous anastomoses are created above the mesentery with no tension. In cases of undesirable anatomy for the portal drainage, the graft is already prepared for the systemic drainage, while now there is no possibility of Y-graft kinking. If the portal drainage is adopted, an extra anastomosis is required. Still, we prefer the procedure due to its promising advantages including shorter ischemic time, lower organ manipulation and less tension.

Another difficulty of the Y-graft and extension technique is the need for an extra conduit. As a significant length of the donor’s iliac artery is preserved for the liver, the problem is solved by taking the required vascular conduit from donor’s carotid artery.

Our results showed the incidence of pancreatitis and rejection was lower in the new Y-graft and extension procedure compared to traditional Y-graft technique. We assume that the mentioned results occurred due to less graft manipulation. As previous studies have reported a low tolerance of pancreas graft to the manipulation [14]. Consequently, episodes of pancreatitis and graft rejection are common issues after pancreas transplantation [15]. A valued benefit of the Y-graft and extension technique is less manipulation of the pancreas and therefore, less complications.

## Conclusion

Post-operative complications tend to be lower in the novel arterial reconstruction technique (Y- graft and extension), however a study on a larger patient group is encouraged to confirm our primary results.

## Data Availability

All data supporting the results of this article are included in this article. Any materials or databases generated in this study are available from the corresponding author upon reasonable request.

## Abbreviations

ASPDA: Anterior Superior Pancreaticoduodenal Artery
ATG: Anti-Thymocyte Globulin
CHA: Common Hepatic Artery
CIA: Common Iliac Artery
DM: Diabetes Mellitus
DPA: Dorsal Pancreatic Artery
IVC: Inferior Vena Cava
PSPDA: Posterior Superior Pancreaticoduoenal Artery
PTA: Pancreas Transplantation Alone
SMV: Superior Mesenteric Vein
SPK: Simultaneous Pancreas Kidney
